# A knowledge-poor approach to chemical-disease relation extraction

**DOI:** 10.1093/database/baw071

**Published:** 2016-05-17

**Authors:** Firoj Alam, Anna Corazza, Alberto Lavelli, Roberto Zanoli

**Affiliations:** ^1^Department of Information Engineering and Computer Science, University of Trento, Italy; ^2^Department of Electrical Engineering and Information Technologies, University of Napoli Federico II, Napoli, Italy; ^3^Center for Information and Communication Technology Fondazione Bruno Kessler, Trento, Italy

## Abstract

The article describes a knowledge-poor approach to the task of extracting Chemical-Disease Relations from PubMed abstracts. A first version of the approach was applied during the participation in the BioCreative V track 3, both in Disease Named Entity Recognition and Normalization (DNER) and in Chemical-induced diseases (CID) relation extraction. For both tasks, we have adopted a general-purpose approach based on machine learning techniques integrated with a limited number of domain-specific knowledge resources and using freely available tools for preprocessing data. Crucially, the system only uses the data sets provided by the organizers. The aim is to design an easily portable approach with a limited need of domain-specific knowledge resources. In the participation in the BioCreative V task, we ranked 5 out of 16 in DNER, and 7 out of 18 in CID. In this article, we present our follow-up study in particular on CID by performing further experiments, extending our approach and improving the performance.

## Introduction

Manual curation of chemical-disease relations (CDRs) from the literature is expensive and it is difficult to keep up with the growing amount of relevant literature. Hence, automatic CDR extraction is of high interest for its potential practical application as an aid for curators. However, the task of curation presents a few characteristics that do not make the adoption of standard relation extraction (RE) approaches a straightforward task, like e.g. Ref. (33). In natural language processing (NLP), RE usually requires considering the mentions of given entities in the document, and to decide whether two specific mentions are connected by a relation. On the other hand, typical curation applications only need to know whether a given text mentions some entities and whether the text supports the existence of a given relation between them.

In the spirit of better matching the actual requirements of practical applications, we decided to approach the tasks in the CDRs track at BioCreative-V, which are different in a few respects from the usual named entity recognition (NER) and RE tasks.

The first difference concerns the ability of the systems to return results within fixed time constraints. Participants were required to setup a web service that was queried by the organizers. This forced the participants to implement a complete system (instead of different, manually connected modules, as it often happens in similar competitions).

In regard to the NER task, recognizing diseases and chemical entities is quite different from annotating more usual entities such as proper names of person (e.g. John Smith) and places (e.g. New York). In fact, chemicals can consist of long multiword expressions (e.g. N-[4-(5-nitro-2-furyl)-2-thiazolyl]-formamide) with large spelling variability (e.g. 10-Ethyl-5-methyl-5,10-dideazaaminopterin vs 10-EMDDA) that requires particular adaptations to the existing methods for NER. In addition, using information like the initial letter capitalization, which proved useful for identifying proper names, cannot be successfully used with diseases and chemical entities given that they often appear in lowercase letters in the text (e.g. nephrolithiasis, triamterene).

As for the RE task, there are two additional crucial differences. First, the entities involved in a relation may appear in separate sentences (according to the task organizers, this happens at least one-fourth of all cases). Second, in the data set the relations are specified making reference to the entities (i.e. their IDs) and not to the mentions of the entities. These characteristics pose some challenges and require a different approach with respect to the ones usually adopted in the literature on RE.

As mentioned above, the chemical-induced disease (CID) task is assessed at the level of the entities in the entire document and not at the level of the specific mentions. This required the adaptation of standard RE approaches to the specificities of the task.

Over the years, a wide variety of RE approaches have been proposed for identifying drug side effects. They applied different strategies: co-occurrence-based statistics, (2, 3) pattern-based approaches (4); machine learning approaches (5) and knowledge-based approaches.

In 2015, the *Journal of Biomedical Informatics* published a Special Issue on Mining the Pharmacovigilance Literature (6). The 13 articles appearing in the special issue establish the state of the art regarding NLP systems and resources related to pharmacovigilance. Among this material, we are interested in the research concerning three tasks, i.e. text classification, NER and RE, when applied to the detection of Adverse Drug Reactions and Drug-Drug Interactions.

Current RE research has been mostly focused on intra-sentential relations, i.e. relations holding between entities appearing in the same sentence. The motivation behind such a choice is that usually the vast majority of the relations involves entities appearing both in the same sentence. This is confirmed by the few papers discussing cross-sentential relations (i.e. relations involving entity mentions beyond sentence boundaries) (7, 8). For example, in Ref. (8) the authors report that 90.6% of the total number of relations in the ACE03 corpus (a RE benchmark in the news domain) are intra-sentential. The authors in Ref. (9) describe a support vector machine (SVM)-based approach to RE that is applied to both intra-sentential and inter-sentential relations.

One of the approaches to address inter-sentential relations consists in the use of co-reference resolution algorithms. For example, this approach was adopted by one of the participants in the CID task (10).

An aspect characterizing the different approaches is the quantity and the nature of the considered *a* *priori* knowledge. In fact, in specific domains, including the biomedical one, domain knowledge is essential for the overall system performance. In our case, for example, as the entities we are looking for consist of chemicals and of diseases, and only relations between such pairs are of interest, the system needs to know possible diseases and chemicals to perform the task. Although this information could in principle be extracted from the training set, a really huge amount of data would be required. On the other hand, resources containing such information are expensive, and cannot be available for every possible task. A workable trade-off should therefore be found, depending on the task at hand and on the available knowledge sources.

In the BioCreative competition, several resources have been exploited by the best-performing systems. As for disease named entity recognition and normalization (DNER), the best performing system (11) exploited NCBI Disease corpus and MEDIC; the second best (12) used medicine’s medical subject headings (MeSH) and Disease Ontology; the third (13) benefitted from MeSH, Disease Ontology, OMIM, Comparative Toxicogenomics Database (CTD) and UMLS. Other domain-specific resources included MedDra, Snomed-CT, ICD10-CM, JoChem, PubChem. Concerning CID, the best performing system (14) exploited CTD, MEDI and SIDER; the second (15) made use of BRAIN (a database containing entities and relations from curated structured databases and Medline texts for almost every concept in UMLS); the third (12) was based on patterns developed manually, requiring an expensive adaptation to the task. In general, it is therefore interesting to analyse the performance of a system exploiting as little external knowledge as possible, in order to assess how far we can get without additional resources. Such knowledge-poor strategy characterizes not only our system, but also the system that ranked fourth (10) which only considered CTD.

A first version of the system described in this article (16) has been presented at the BioCreative V workshop. Starting from that work, we describe here different ways that we explored to improve it:

1. First of all, we fixed a few bugs, and obtained a better performance.
2. We added three new features, which produced a further performance improvement.
3. We introduced word-embedding features.
4. We integrated the classifier designed on the whole abstract with a sentence level classifier and tried four different strategies to integrate the two outputs.

In the following section, we discuss more in detail the specificities of the task we are considering. System architecture section is devoted to the description of the approach, by considering all different modules. In experiments section, we present the experimental assessment of all the variants of the system. A final section discusses the obtained results and possible future research directions.

## Task description

In this section, we briefly present the BioCreative V track 3 (17, 18), from which the approach described in the article originates. The task consists of the automatic extraction of (CDR) from PubMed articles. It includes two subtasks: DNER and CID RE.

The data set consists of 1500 PubMed abstracts randomly selected from the CTD-Pfizer corpus (1400 articles) and from a new set of curated articles (100 articles). The CTD-Pfizer corpus consists of over 150 000 chemical-disease relations in 88 000 articles (19, 20). For the CDR task, the organizers split data into training, development and test sets with 500 articles in each set. They annotated the data set with diseases and chemicals using PubTator tool (21) and facilitated human annotation with automatic systems such as DNorm (22) and tmChem (23). The annotation includes both the mention text spans and normalized concept identifiers. The concept identifiers are defined using the National Library of MeSH controlled vocabulary. The entities were annotated independently by two annotators. The average inter-annotator agreement scores are 88.75% and 96.31% for the disease and chemicals, respectively, in the test set according to the Jaccard similarity coefficient (24).

The DNER task consists of recognizing and normalizing Diseases, which is an intermediate step for the automatic CDR extraction. For this task, participating systems were given raw PubMed abstracts as input and asked to provide normalized disease concept identifiers.

The CID task includes finding the chemical-induced disease relations. For this task, the same input (i.e. raw PubMed abstracts) was used and the systems were asked to return a list of <chemical, disease > pairs with normalized concept identifiers for which chemical-induced disease are associated in the abstract.

In Table 1, we present some figures about the data set. In both training and development set the distribution of chemicals and diseases is around 55% and 45%, respectively. In the last two columns of Table 1, we present the number of mentions and the number of associated entities (within parenthesis) for chemicals and diseases respectively. More details on the task can be found in Refs (17) and (18).

**Table 1:** Summary of the BioCreative V track 3 data set

Data set	No. of. doc.	No. of rel.	No. of unique rel.	No. of avg. token per doc.	No. of avg. token per title.	No. of Avg. token per abs.	No. of chemical mention (ID)	No. of disease mention (ID)
Train	500	1039	928	216.75	13.52	203.23	5203 (1467)	4182 (1965)
Dev	500	1012	889	215.33	13.61	201.72	5347 (1507)	4244 (1865)
Test	500	1066	941	226.57	13.42	212.59	5385 (1435)	4424 (1988)

Final evaluation of the participants’ systems was performed by comparing their output against manually annotated entities and relations using precision, recall and *F*_1_. DNER results were evaluated by comparing disease concepts only, whereas CID results were evaluated by comparing chemical-disease relations.

## System architecture

**Figure 1:**
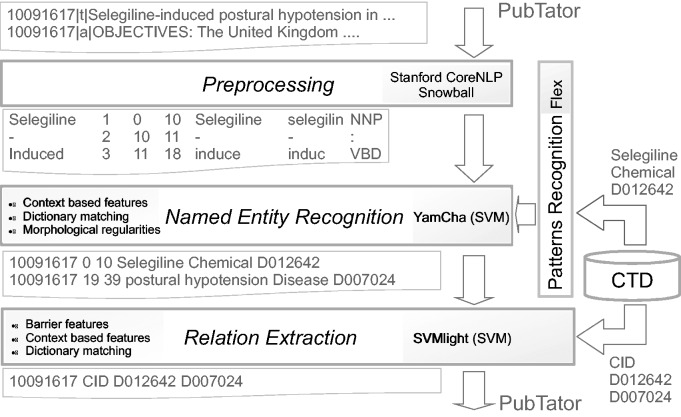
System architecture.

For both tasks, we have adopted a general-purpose approach using freely available tools for preprocessing data. While the CID step is based on a machine learning approach, DNER combines machine learning and pattern matching. As a design choice, the system only uses the data sets provided by the organizers. We preprocessed the data set with the Stanford CoreNLP pipeline that extracted the base forms of words, their parts of speech, and performed sentence segmentation. For the DNER task, the features include knowledge extracted from the CTD, morphological regularities obtained by extracting prefixes and suffixes of the words, and context-based features extracted in a local context where the entities appear. The implemented system recognizes both diseases and chemical entities. For the CID task, our approach extracts features from the CTD along with other linguistic features. Different feature configurations have been compared. For the official submission the configuration using lemma and stop word filtering was chosen. Figure 1 shows the system architecture. In the following, we provide the details of each step.

### Preprocessing

We use Stanford CoreNLP (http://nlp.stanford.edu/software/corenlp.shtml) (25) to obtain the base form of the words, their part of speech (POS) and lemma, and to perform sentence segmentation. The Snowball tool is used for producing the stem of the words (http://snowball.tartarus.org/).

### Comparative toxicogenomics database

As a domain-specific resource we have exploited the CTD (20), a publicly available database that aims to advance understanding about how environmental exposures to chemicals affect human health. It provides manually curated information about chemicals, and diseases that, in our approach, are used to capture the different ways the entities are mentioned in texts. During the preprocessing, chemical and disease terms (names, symbols and synonyms) are first extracted from the database, and then converted into regular expression patterns. In this way, we extracted 533 646 regular expressions for Chemicals and 92 024 for Diseases, which we used for matching 1138 Chemicals and 876 Diseases in the training set. After that, we use Flex, a tool for generating programs which recognize lexical patterns in text (http://flex.sourceforge.net/). Flex generates the scanners to recognize the mention patterns which are used further for training the classifier. We also use the chemical-disease relationships database. It includes chemical-disease pairs and it has been exploited in the CID subtask to know the entities in texts that have a relation in the CTD.

### Named entity recognition

DNER is performed in two steps: (i) detecting the mentions of the entities in text (mention detection) and (ii) selecting the best-matching MeSH ID (normalization).

‘Mention detection’ is complex because an entity can appear in texts in many different ways. For example, ‘acetylsalicylic acid’ could be reported using the systematic nomenclature (typically multiword terms with large spelling variability), describing the compound in terms of its structure (i.e. ‘2-(Acetyloxy)benzoic acid’), rather than non-systematic nomenclature (i.e. ‘aspirin’) or synonyms like ‘acetylsalicylate’. To classify mentions we combine three approaches:

- ‘Dictionary matching’ consists in finding a mention in text by comparing it with a dictionary. We use the scanners generated by Flex during the preprocessing to recognize both Chemicals and Diseases.
- ‘Exploiting morphological regularities’ is done by using the prefixes and suffixes of the tokenized words, and the stem of the word. The suffix *-emia* is, for example, typical of diseases (e.g. ischemia), whereas the prefix *meth-* is useful for chemicals discrimination (e.g. methylxanthine)
- ‘Context-based features’ are implemented by considering a window of length 4 consisting of the current token, one token before and two tokens after.

Such approaches are combined by means of YamCha, an open source customizable text chunker based on SVMs (http://chasen.org/taku/software/yamcha/). With YamCha it is possible to redefine the feature sets (window-size) and we considered whether or not the token matches with the vocabulary. The system also considers the POS of the token before the current token, the prefixes/suffixes of the two following tokens, and the entity labels assigned during the tagging to the two tokens before.

‘Normalization’ selects the best-matching MeSH ID by means of ‘dictionary matching’ based on CTD (see the pseudocode in Algorithm 1).

Algorithm 1. Pseudocode for Mention Normalization. pred_mentions are the mentions recognized by the NE system. gold_mentions, ctd_chemical, ctd_disease are dictionaries in which mentions are associated with MeSH IDs

**Input:** gold_mentions,ctd_chemical,ctd_disease,pred_mentions

**Output:** normalized_mentions

**procedure** *normalization*(gold_mentions,ctd_chemical,ctd_disease,pred_mentions)

** for all** $mention_{i}\in pred\_mentions$ **do**

**  if** $mention_{i}\in gold\_mentions$ **then**

**   **$mention_{i\_id}\leftarrow gold\_mentions.getMostFrequentID(mention\_i)$

**  else if** $mention_{i}=chemical\&mention_{i}\in ctd\_chemical$ **then**

**   **$mention_{i\_id}\leftarrow ctd\_chemical.getMostFrequentID(mention\_i)$

**  else if** $mention_{i}=disease\&mention_{i}\in ctd\_disease$ **then**

**   **$mention_{i\_id}\leftarrow ctd\_disease.getMostFrequentID(mention\_i)$

**  else**

**   **$mention_{i\_id}\leftarrow -1$

**  end if**

** end for**

**end procedure**

One of the major issues of the normalization task is the ambiguity between identifiers that happens when one mention refers to many identifiers (e.g. psychosis Disease was identified six times with D011618 and twice with D011605 in the training set). However, with regard to our specific task, case reports of this phenomenon are rare, with only five cases observed in the training set. Our method addresses this problem by returning the identifier that has been most frequently associated with the given entity in the training set (e.g. D011618 is the identifier assigned to psychosis). The same approach was also used to map the mentions in texts with the terms extracted from CTD.

Finally, it is worth mentioning another problem that often comes up with Named Entity Recognition in biomedical texts, and that requires to identify and resolve composite named entities, where a single span refers to more than one concept (e.g. neurological and cardiovascular toxicity). In this regard, only 1% of disease and chemical mentions are composite mentions in the provided data set, and so we do not use any specific resource (e.g. SimConcept tool) to deal with such cases.

### Relation extraction

As mentioned above, it is not straightforward to apply standard RE approaches in the CID RE task due to the specific characteristics of the task. In NLP, the relations are usually annotated at the level of the mentions of the entities involved and they connect entities appearing in the same sentence. In CID both limitations do not hold. First, the relations are annotated at the level of the entire abstract, involving entities (and not specific mentions). Second, the relations may involve entities not appearing in the same sentence (∼25% of the annotated relations in the data set). These characteristics require an approach that combines two interconnected perspectives. On one hand, a binary classification task considers the whole abstract, taking as input a pair of entities, and gives a positive output when the two entities are in relation. On the other hand, from the perspective usually applied in the NLP field, a relation is realized between pairs of mentions rather than between pairs of entities, and only involves the sentence where the two mentions appear rather than the entire abstract.

Therefore, we could not limit our analysis to sentences in isolation. However, considering larger chunks of text would imply including a much larger set of mention pairs, and therefore a considerable increase in computational effort. We therefore decided to limit the latter perspective to the single sentence: we refer to it as Sentence Level Classifier (SLC), and always consider its integration with a Document Level Classifier (DLC), which involves entity pairs in the entire abstract. The goal of the DLC consists in determining whether the abstract states that two given entities are connected by a relation. Each entity can be represented by one or more mentions occurring in the text.

### Features

Classification is performed in a Vector Space Model, where the Feature Vector (FV) corresponding to each potential relation is constructed by the juxtaposition of the FVs corresponding to the two entities, together with a set of ‘relation features’, which take into account both entities. In this way, the classifier directly decides whether a relation exists between the two entities.

In the first version of the system that participated in the BioCreative V CDR task (16), we only considered a DLC, which takes such a FV built from the abstract for every pair of chemical and disease entities. As each entity is associated with one or more mentions, we define a FV for each mention, and then combine them by OR operation to obtain the FV of the entity. On the other hand, each relation is linguistically realized between two mentions rather than between the two entities. Therefore, we want to integrate the DLC with another one, which considers every pair of mentions compatible with a relation and occurring inside one sentence. Although we cannot consider this alone, because in the training set about one-fourth of all relations connect mentions occurring in different sentences, we want to check whether such a classification can help to improve the performance.

All in all, we therefore consider a FV for each mention and then combine them in a FV for each entity for the DLC while we consider them separately for the SLC. All the features considered here are Boolean, and mention FVs are combined by means of an OR operation into entity FVs. Again, each mention FV is built by considering the OR of each token FV, which are based on a set of Boolean features that signal the occurrence of given patterns in the token. These features include:

- the first and the last characters,
- word prefixes and suffixes of length from 3 to 5,
- whether the first character is a capitalized letter, an uncapitalized letter, or a number,
- whether the word contains one or more uncapitalized letters or is only composed by capitalized or only by uncapitalized letters and
- whether the token contains a dot, a comma or a hyphen.

A set of features is also represented by word and POS unigrams, bigrams and trigrams from a window of length five centered in the token (i.e. the current token, two tokens to its left and two tokens to its right).

In addition to these features, in some configurations of our system we also considered Barrier Features (BFs) (26), which are based on the set of trigger POS tags and corresponding endpoints listed in Table 2. POS tags are taken from the Penn Treebank tag set. Given a token whose POS tag corresponds to a trigger, we consider the closest token at its left having the endpoint POS tag. The set of POS tags included between them is collected, and each BF is defined by the following triple: trigger, endpoint and set of included tags.

**Table 2:** Trigger and endpoint pairs for barrier features

Endpoint	Trigger
JJ	JJR
DT	NN, NNP
PRP	NNS
JJ	RBR
DT, IN	VB
IN	VBP
DT, MD, VB, VBP, VBZ, TO	VBD, VBN
PRP	VBZ

Moreover, by following the indications in (14), we completed the representation of each entity involved in the relation with the following three features:

1. Does the Chemical appear in the title of the document?
2. Does the Disease appear in the title of the document?
3. Is the Chemical a Core Chemical (i.e. either it is the most frequent in the document or it appears in the title)?

Last, but not least, we also included ‘word-embedding features’, which have recently been very popular in several NLP tasks. Word embeddings, also known as context predictive model or neural language model, are new techniques to design distributional semantic models (DSMs), which differ from traditional DSMs where co-occurrence counts are used (27). In word embedding, distributed vector representations are learned from a large corpus by neural network training, and represent them in a low dimensional continuous space. It has been proven that such representation better capture semantic and syntactic relationships (28).

To design word-embedding models, we collected full-text of articles from the PubMed Central Open Access section (ftp://ftp.ncbi.nlm.nih.gov/pub/pmc/), till the 24 October 2015, containing 1 35 7 967 articles. Part of the articles is in *nxml* format, which we converted into raw texts using a specific tool (https://github.com/spyysalo/nxml2txt). The raw texts are then sentence splitted and tokenized using the Stanford CoreNLP tool to prepare the data for designing the word-embedding model. We utilized word2vec toolkit for training (https://code.google.com/p/word2vec/), which is an implementation by Mikolov *et al.* (29), and contains both continuous bag-of-words (CBOW) and skip-gram algorithms. We designed our model using the CBOW approach with a size of the FV 500, a context window size 5, negative-sampling with a value of k = 10. The resulting trained word-embedding model contains 6 billions words with a vocabulary of size 1.8 millions.

Differently from the other features, word embeddings are composed by real numbers. We used two different approaches to obtain features for each relation example. The first approach computes similarity between the sets of words respectively referring to chemical and disease by utilizing the word-embedding model’s similarity measure and then uses it as a feature. In this way, we added a new feature evaluating how much the two entities are similar when considered by word embeddings. The rational under this is that two related entities will be more similar than unrelated ones.

In the second approach, we call *C* and *D* the set of different words occurring in chemical and disease mentions respectively and we define a set of words by T=C∪D, for the entity pair of a training example. Then, we extract the FV for each word from the word-embedding model. After that we compute the average of word vectors to have an equal sized vector for each training instance. For example, if we have a set of 10 words of a chemical-disease relation then we obtain a matrix of size 10 × 500. By taking an average we obtain a FV of size 1 × 500. Using this approach we add 500 features.

Our approaches towards utilizing the word-embedding features are in a very early stage. Since word embeddings have recently proven to be useful in different NLP tasks, we plan to try different feature representations to feed into the classifier as a future work.

All features considered above regard the entities. As mentioned above, we also consider four binary relation features, depending on both entities, defined as follows:

1. Is the entity pair listed as a positive chemical-disease relation in the CTD (20)?
2. Do the mentions of both entities appear in the same sentence?
3. Do the mentions of both entities appear in the same sentence in the title?
4. Do the mentions of both entities appear in the same sentence in the abstract?

Note that the first one is the only feature based on an external knowledge source. As relation features are more likely to predict the existence of an actual relation, we overweigh them with respect to entity features by introducing a relation features weight (RFW) greater than 1.

‘Feature selection’ is needed because of the potentially very large number of *n*-grams and BFs. To address this issue, we prune all features not occurring or occurring with not enough instances in a different data set. As such feature selection strategy is more effective when the external data set well describes the task at hand, we consider the corpus used for the BioCreative IV Chemical compound and drug name recognition (CHEMDNER) task (30), consisting of 27 000 documents (both titles and abstracts). The threshold to decide which features to prune is set to the mean of all counters. This results in a feature set of 102 297 features.

### Classification

Classification is performed using SVMlight (http://svmlight.joachims.org/) (31). Note that this classifier associates a margin to every output: if the margin is positive, the output will be positive, and negative in the opposite case. The larger the absolute value of the margin, the more the classification decision is reliable.

Moreover, we apply a post-processing phase on the output of the automatic RE system. The goal of the post-processing is to increase recall, at the cost of some degradation in precision. As a preliminary step, we build a chemical-induced disease relation dictionary using the positive examples in the training and development data. In the post-processing phase, we first check whether any entity pair in the test set is identified by the automatic RE system or not. If not, then, we use the CID relation dictionary and check if the candidate entity pair matches an entry in the dictionary. If there is a match, then we label such an entity pair as a positive example.

### Combination of the basic classifiers

At this point we have a single DLC and several SLCs, one for each entity pair which is compatible with the relation, and we want to combine them to obtain the complete classification. First of all, we can separately consider the two levels: the DLC directly produces the requested classification, while we can combine the SLCs with an OR operation, that is the classification is positive whenever at least one SLC gives a positive response. However, this combination of SLCs would miss all cross-level relations, which were about the 25% in training data. We therefore try to combine the two level classifiers together.

The most straightforward strategy for such a combination is to consider the OR of the outputs of the two classifiers. In other words, we consider a relation as positive whenever either the DLC or the SLC does so. We refer to this strategy as *S*_1_. Another possibility involves adding the output of the SLC as a feature for DLC: this is *S*_2_. The last two strategies consider a linear combination of the output of the two classifiers. While *S*_3_ takes equal weights for the two levels, *S*_4_ considers the number *p* of SLCs returning a positive margin: in this case the weight associated to the DLC is given by $\frac{1}{p+1}$, and therefore the one associated to the SLC is $\frac{p}{p+1}$.

Even in cases where we have a constraint on execution times, as was the case in the BioCreative V workshop, this approach is viable because the two classifiers can be run in parallel and all the four combination strategies we consider require a very small overhead.

## Experiments

During the official participation in the task, to find the best system configuration and to assess the system performance, we trained both the systems for DNER and CID on the provided training data set and tested it on the development set. For the official submission, we trained both the systems for DNER and CID on the training and development sets and applied them to the test set.

The experiments performed after the official submission (and the release of the gold standard test set) have been performed directly training the system on the training and development sets and applying the learned model to the test set.

In Tables 3 and 4, we present a brief overview of the different experimental strategies/settings used for DNER and CID tasks. More details of these strategies/settings can be found in the next two sections.

**Table 3:** Different experimental strategies of the DNER task, including with/without external resources and feature analysis

Strategy	Description
Default configuration	Dictionary matching (CTD) + morphological regularities + context based features
Baseline#1_CTD	Dictionary matching (CTD) only
Baseline#2_w/o_res	ML system on the training set without any additional resource
−Dictionary matching (CTD)	w/o dictionary matching
−Context-based features	w/o context-based features
−Morphological regularities	w/o morphological regularities

**Table 4:** Different experimental strategies of the CID task, including feature level analysis and classifier combinations

Strategy	Description
DLC	Entity pair in the entire abstract
SLC	Entity pair within a single sentence
Combo (*S*_1_)	OR of the outputs of the two classifiers DLC and SLC
Combo (*S*_2_)	The output of SLC is added as a feature for DLC
Combo (*S*_3_)	Linear combination of the output of the two classifiers with equal weights
Combo (*S*_4_)	Linear combination of the output of the two classifiers with weights computed as in combination of the basic classifiers
Basic feats	Features of the two entities + binary relation features
All-feats	Added three new features (Chemical in title; Disease in title; Core Chemical)
BFs	(see Features)
Word embeddings	(i) 1 feature (ii) 500 features (see Features)

### Disease named entity recognition and normalization

Given that the data set contains annotations for both chemical and disease entities, we have implemented a single system for recognizing both the entity types in the DNER and CID subtask even though DNER does not require it. Table 5 reports the results of chemical-disease mention detection and normalization on the development set. The results were obtained by the default configuration of the system described in named entity recognition, and compared with two baselines: baseline#1_CTD is calculated by matching the chemical and disease mentions in the texts with the CTD and by normalizing them with the MeSH ID associated to those mentions in the CTD; baseline#2_w/o_res is calculated by training the system on the tokenized articles in the training set without any additional source of information. Finally, we retrained the system on the training set plus the development set and evaluated it on the test set. The results obtained are shown in Table 6. In this regard we ranked 5 out of 16 participants.

**Table 5:** Results of entity normalization and mention detection (in brackets) on the development set

	P	R	F1
Chemical	88.11(92.24)	88.05(86.95)	88.08(89.51)
Disease	84.31(83.50)	77.57(80.75)	80.80(82.10)
Chemical+Disease	86.09(88.32)	82.26(84.20)	84.13(86.21)
baseline#1_CTD	76.03(81.07)	64.01(69.47)	69.51(74.82)
baseline#2_w/o_res	88.14(78.40)	64.13(64.21)	74.24(70.60)

**Table 6:** Results of entity normalization and mention detection (in brackets) on the test set

	P	R	F_1_
Chemical	88.57(93.50)	88.57(89.71)	88.57(91.57)
**Disease**	86.82(84.15)	81.84(82.21)	84.26(83.17)
Chemical+Disease	87.58(89.24)	84.66(86.33)	86.09(87.76)

In bold the system’s official results

To measure the impact of the different sources of information on the final system performance on the development set, we removed one type of information at a time from the system default configuration. Table 7 reports these results.

**Table 7:** Variation in results of entity normalization and mention detection (in brackets) when we remove one type of information at a time

	P	R	F_1_
Chemical+Disease entities			
−Dictionary matching (CTD)	+1.84(−1.32)	−17.62(−5.95)	−9.62(−3.81)
−Context-based features	−3.46(−9.41)	−0.3(−2.26)	−1.84(−5.82)
−Morphological regularities	−1.63(−0.43)	+0.59(−0.23)	−0.43(−0.48)
Disease entities			
−Dictionary matching (CTD)	+0.89(−2.66)	−13.94(−4.57)	−7.95(−3.66)
−Context-based features	−2.53(−10.53)	−0.75(−4.03)	−1.58(−7.30)
−Morphological regularities	−0.89(−0.39)	+0.70(−0.61)	−0.04(−0.50)

### Chemical-induced diseases relation extraction

As for the CID subtask, we compared the performance of different configurations on the test set, after training on the union of training and development sets. The data set is characterized by a strong unbalance between positive and negative items. To address the data set skewness, we optimized the cost-factor parameter on the development set (see Ref. (16)). As a result of such optimization, in the official submission, we considered a cost-factor of 4.3. Furthermore, we set RFW to 5, apply lemmatization to deal with data sparseness and consider the linear kernel for SVM. As for the choice whether to use BFs and a list of stopwords, the mentioned preliminary experiments showed that all four possible settings perform very similarly. For the sake of both efficiency and robustness, we therefore tried to minimize the number of features and introduced the stopword filtering, but not the BFs.

In Table 8, we present a performance comparison of our system with the one built for the official competition (16), which consisted of a DLC only. Of course, a direct comparison is meaningful only considering the performance with Automatically Recognized Entities (ARE). To assess the performance of the RE system *per se*, we also report performance with Gold Standard Entities (GSE). Although the system labelled as ‘basic feats’ in the table is actually the same used for the task participation, performance is a bit better because after the official submission at the competition, we fixed a few bugs in the software. The new system is labelled ‘all-feats’ to underline the fact that we added three new features, as discussed in system architecture. These new features produced a further improvement in performance, both when GSE are considered and in the more realistic case when they have been automatically recognized.

**Table 8:** Results of different configurations of the RE system

	Document-level			Sentence-level			Combo (*S*_1_)		
	P	R	*F*_1_	P	R	*F*_1_	P	R	*F*_1_
GSE									
Basic feats	42.41	77.39	54.79	47.96	56.37	51.83	40.33	80.30	53.70
All-feats	44.18	79.08	56.69	49.47	57.22	53.06	43.05	80.01	55.98
ARE									
BioCreative V	35.39	56.47	43.51						
Basic feats	37.98	61.06	46.83	52.01	19.41	28.27	37.54	61.81	46.72
All-feats	40.31	63.03	49.17	53.94	19.23	28.35	40.14	63.22	49.10

Basic features: the ones used for the official submission; All features: includes the three new features (Chemical in title; Disease in title; Core Chemical).

The results obtained by the inclusion of word-embedding features can be evaluated by considering the performance reported in Table 9 and comparing them with those reported in Table 8. Not only the performance of the two versions of these features is very similar, but it is also very similar to those obtained without such features.

**Table 9:** Results with word-embedding features

	Document-level			Sentence-level			Combo (*S*_1_)		
	P	R	*F*_1_	P	R	*F*_1_	P	R	*F*_1_
GSE									
Chemical-Disease similarity (1 feature)	44.66	79.55	57.20	49.35	56.85	52.83	43.30	80.39	56.29
Average of the FVs of words (500 features)	44.79	79.36	57.26	49.35	57.04	52.92	43.68	80.39	56.61
ARE									
Chemical-Disease similarity (1 feature)	39.65	63.60	48.85	53.79	19.32	28.43	39.47	63.79	48.76
Average of the FVs of words (500 features)	39.89	63.13	48.89	53.75	19.51	28.63	39.67	63.23	48.75

The second block of columns in Table 8 reports the performance of the SLC, which has been newly introduced in the work presented in the article. Sentence level classification remarkably improves precision, both with basic and complete features and both with GSE and ARE. Unfortunately, this occurs at the cost of a larger decrease in recall, so that the *F*_1_ value is always worse than for the DLC. This difference in *F*_1_ between DLC and SLC is impressively larger for ARE.

The third block of columns in the same table reports the performances of the combinations of DLC and SLC with strategy *S*_1_. It should be considered together with Table 10 where the different combination strategies introduced in system architecture are reported for the system with the complete feature set. Note that the first line of this table reports the same numbers reported in Table 8. Also in this case we obtain two different patterns with GSE and ARE: in the former case, the strategy which performs better is the linear combination of the two outputs with equal weights (*S*_3_), while with ARE the OR combination (*S*_1_) should be preferred. In both cases, however, the performance of strategy *S*_2_ is really bad, even worse than SLC alone. In this case, the output of SLC classifier is given as input to the DLC: evidently it pushes the performance to a behaviour similar to the SLC.

**Table 10:** Results of different combination strategies of the two classifiers

	P	R	*F*_1_
GSE			
*S*_1_	43.05	80.01	55.98
*S*_2_	43.18	50.84	46.70
*S*_3_	44.39	76.92	56.29
*S*_4_	43.05	80.01	55.98
ARE			
*S*_1_	40.14	63.22	49.10
*S*_2_	51.12	16.97	25.49
*S*_3_	24.49	70.63	36.37
*S*_4_	24.39	71.01	36.31

For the sake of a deeper analysis of the experimental results, we also report the absolute number of errors, distinguishing between False Negatives (FNs), that is, the number of relations in the ground truth which have not been detected by the classifiers (Table 11), and False Positives (FPs), that is the number of spurious relations introduced by the system (Table 12). We separately consider the two classifiers, SLC and DLC. Note that the data presented in Tables 11 and 12 do not depend on any post-processing phase (differently from the performance in the previous tables). This has been done in an effort to better assess the effect on FNs and FPs and separate it from the effect of the post-processing phase.

**Table 11:** Number of FNs in the results of the two classifiers

	Tot	Same sent.	Other
GSE			
DLC	261	96	165
SLC	551	183	368
ARE			
DLC	431	216	215
SLC	947	579	368

**Table 12:** Number of FPs in the results of the two classifiers

	Tot	Same sent.	Other
GSE			
DLC	1002	671	331
SLC	506	506	0
ARE			
DLC	930	242	688
SLC	96	96	0

The DLC tends to be propositive, meaning that the number of FNs it introduces is much lower than the number of FPs, while the SLC is much more conservative, and introduces fewer FPs with respect to the number of FNs.

Furthermore, in both tables we separately consider the number of errors due to relations connecting entities in the same sentence. As all hypotheses generated by the SLC are of this type, only FPs involving entities within the same sentence are introduced by this classifier. On the other hand, it misses all the relations which do not involve entities appearing in the same sentence, and therefore the number of FNs of this kind does not depend on the fact that we are considering the GSE or ARE case.

In general, we note relevant differences between the performance with GSE and with ARE. To better analyse such difference, in addition to the standard performance measures (precision, recall and *F*_1_), we computed ‘pairs completeness’ (32). This measure is particularly relevant when evaluating the performance of the combination of a named entity recognizer and of an RE system. Pairs completeness measures the upper-bound on recall for the RE task, independently from the specific algorithm used for extracting relations. Pairs completeness is defined as the ratio between the number of positive examples produced by our generative procedure and the total number of positive examples in the annotated corpus. Note that pairs completeness can be calculated only if manually annotated relations are available. The value of Pairs Completeness using our DNER system is 78.96.

## Conclusions and future work

We considered different possible improvements on the system presented in the Ref. (16), and in fact the final system has better performance with respect to the one presented there. Further experimentation is required to optimize the choice of the most effective features by means of a composition of feature design and feature selection. Moreover, we plan to apply different approaches as SLC (e.g. the one proposed in the Ref. (33)). Furthermore, syntactic features can help in improving performance. They can be included in the system either as word pairs constructed on the basis of a dependency parsing analysis, or as a complete constituency parsing to analyse by means of a tree kernel SVM (34). Last, but not least, a more sophisticated choice of potential relation candidates among all possible entity pairs can be introduced to help improve the performance. For example, we could introduce an *a priori* probability for each of the candidate pairs. Such *a priori* probabilities can be either extracted from a probabilistic ontology if available or evaluated from data. For example, we could estimate such probability by backing off to super-classes for the two entities.

## Funding

Funding for open access charge: Fondazione Bruno Kessler (Trento, Italy).
